# Non-destructive mid-IR spectroscopy with quantum cascade laser can detect ethylene gas dynamics of apple cultivar ‘Fuji’ in real time

**DOI:** 10.1038/s41598-021-00254-1

**Published:** 2021-10-19

**Authors:** Masaki Yumoto, Yasushi Kawata, Tetsuya Abe, Tomoki Matsuyama, Satoshi Wada

**Affiliations:** 1 Photonics Control Technology Team, RIKEN Center for Advanced Photonics, RIKEN, 2-1 Hirosawa, Wako, Saitama 351-0198 Japan; 2 TOPCON CORPORATION, 75-1 Hasunuma-cho, Itabashi, Tokyo 174-8580 Japan

**Keywords:** Optical spectroscopy, Plant stress responses, Plant sciences, Optical spectroscopy

## Abstract

Many plants, including fruits and vegetables, release biogenic gases containing various volatile organic compounds such as ethylene (C_2_H_4_), which is a gaseous phytohormone. Non-destructive and in-situ gas sampling technology to detect trace C_2_H_4_ released from plants in real time would be attractive for visualising the ageing, ripening, and defence reactions of plants. In this study, we developed a C_2_H_4_ detection system with a detection limit of 0.8 ppb (3σ) using laser absorption spectroscopy. The C_2_H_4_ detection system consists of a mid-infrared quantum cascade laser oscillated at 10.5 µm, a multi-pass gas cell, a mid-IR photodetector, and a gas sampling system. Using non-destructive and in-situ gas sampling, while maintaining the internal pressure of the multi-pass gas cell at low pressure, the change in trace C_2_H_4_ concentration released from apples (*Malus domestica* Borkh.) can be observed in real time. We succeeded in observing C_2_H_4_ concentration changes with a time resolution of 1 s, while changing the atmospheric gas and surface temperature of apples from the ‘Fuji’ cultivar. This technique allows the visualisation of detailed C_2_H_4_ dynamics in plant environmental response, which may be promising for further progress in plant physiology, agriculture, and food science.

## Introduction

Plants generate various phytohormones and substances in their tissues and organs at all growth stages, and their expression can change in response to infectious diseases. Ethylene (C_2_H_4_) is the lightest gaseous phytohormone. It is involved in many functions, such as leaf and flower senescence, promotion of seed germination and flowering, and softening and ripening of fruit^1–4^. Moreover, part of the C_2_H_4_ produced in the plant is released as a volatile organic compound (VOC). For example, ripe apples and avocados release approximately < 200 µL·kg^−1^ h^−1^ of C_2_H_4_^5–8^. Rice releases a low concentration of approximately 10 µL·kg^−1^ h^−1^ of C_2_H_4_ upon disease infection^9^. It has also been reported that soya beans and wheat release 10–60 ppb of C_2_H_4_ during periods of canopy expansion and rapid growth^10^. The amount of C_2_H_4_ released depends on the weight and growing conditions of the plants, and the concentration of C_2_H_4_ is strongly influenced by the gas sampling method. In many cases, non-destructive and real-time detection of ppb-level C_2_H_4_ allows the visualisation of C_2_H_4_ dynamics to plant environmental responses.

Some methods to detect trace C_2_H_4_ exist, such as gas chromatography-mass spectrometry (GC/MS), chemiresistive sensors, and laser sensing. GC/MS is a widely used method for detecting and analysing numerous VOCs, including C_2_H_4_^11,12^. Nevertheless, GC/MS is not suitable for real-time detection of gas concentration changes, owing to the necessity of long measurement times and complex pretreatments. However, chemiresistive sensors made from single-walled carbon nanotubes are now available, which are tiny sensors that allow real-time detection of sub-ppm level C_2_H_4_^13,14^. In addition, artificial metalloenzyme-based chemical biosensors have been used to detect ethylene gas spatially by fluorescence in fruits and leaves^15^. However, it is difficult to detect ppb-level C_2_H_4._ Moreover, these sensors respond to several gases (e.g., ethanol, acetaldehyde, and hexane) other than C_2_H_4_.

Optical sensing technologies based on laser spectroscopy allow rapid detection of trace VOCs. These include photoacoustic absorption spectroscopy (PAS)^16,17^, cavity ring-down spectroscopy (CRDS)^18,19^, and laser absorption spectroscopy (LAS)^20,21^ using tunable diodes or quantum cascade lasers (QCLs). A detection limit of 0.3 ppb (for 2σ) of C_2_H_4_ has been demonstrated with PAS using a CO_2_ laser as the pump source^22^. When a quantum cascade laser (QCL) was used as a pump source, a detection limit of 50 ppb of C_2_H_4_ was obtained, and C_2_H_4_ was successfully detected in the gases released from the apple^23^. In this case, the gas released from the apple was collected in a slightly complicated manner using a sample bag. The apples were placed in the bag, and a pump fully evacuated the air inside the bag. Then, N_2_ with 99.999% purity was introduced into the bag until atmospheric pressure was reached. After waiting for at least 15 min, the gas in the bag was used for the measurement. In CRDS, sub-ppb C_2_H_4_ detection has been realised using a near-IR distributed feedback (DFB) laser oscillating at 1.6 µm^24^. However, these optical sensing methods are not yet robust and user-friendly because they require high-power lasers and optical cavities with precision adjustment.

LAS is highly robust and user-friendly compared to PAS and CRDS, owing to its simple measurement setup. Therefore, LAS is used for various forms of gas absorption spectroscopy, although its detection sensitivity is inferior to that of PAS and CRDS. Recently, using a 1.62-µm DFB laser and a 3.27-µm interband cascade laser, respective detection limits of 6.6 and 53 ppb C_2_H_4_ have been demonstrated^25,26^. However, the detection limits of LAS-based optical sensing systems have not yet reached the ppb level. When detecting C_2_H_4_ released from plants using optical sensing technologies, it is necessary to place the plant into a sample bag or glass chamber to collect gas. Moreover, a carrier gas such as N_2_ must be used. Non-destructive optical sensing technology that uses in-situ gas sampling methods in an open environment would represent an advance in biogenic gas analysis technology for plants. For example, they would allow us to observe rapid changes in plant environmental response and identify the source of VOCs. The resulting technology would be useful for early disease diagnosis and stress susceptibility monitoring in the plant physiology and breeding fields, and would facilitate efficient quality control of fruits and vegetables in the postharvest industry.

In this study, we developed a C_2_H_4_ detection system based on LAS using a QCL and demonstrated the real-time detection of C_2_H_4_ released from apples by non-destructive and in-situ gas sampling in an open environment. When apples were stressed by changes in atmospheric gas concentration or surface temperature, we also succeeded in observing rapid changes in the concentration of C_2_H_4_ released from apples.

## Results and discussion

### Trace gas detection system

**Figure 1:**
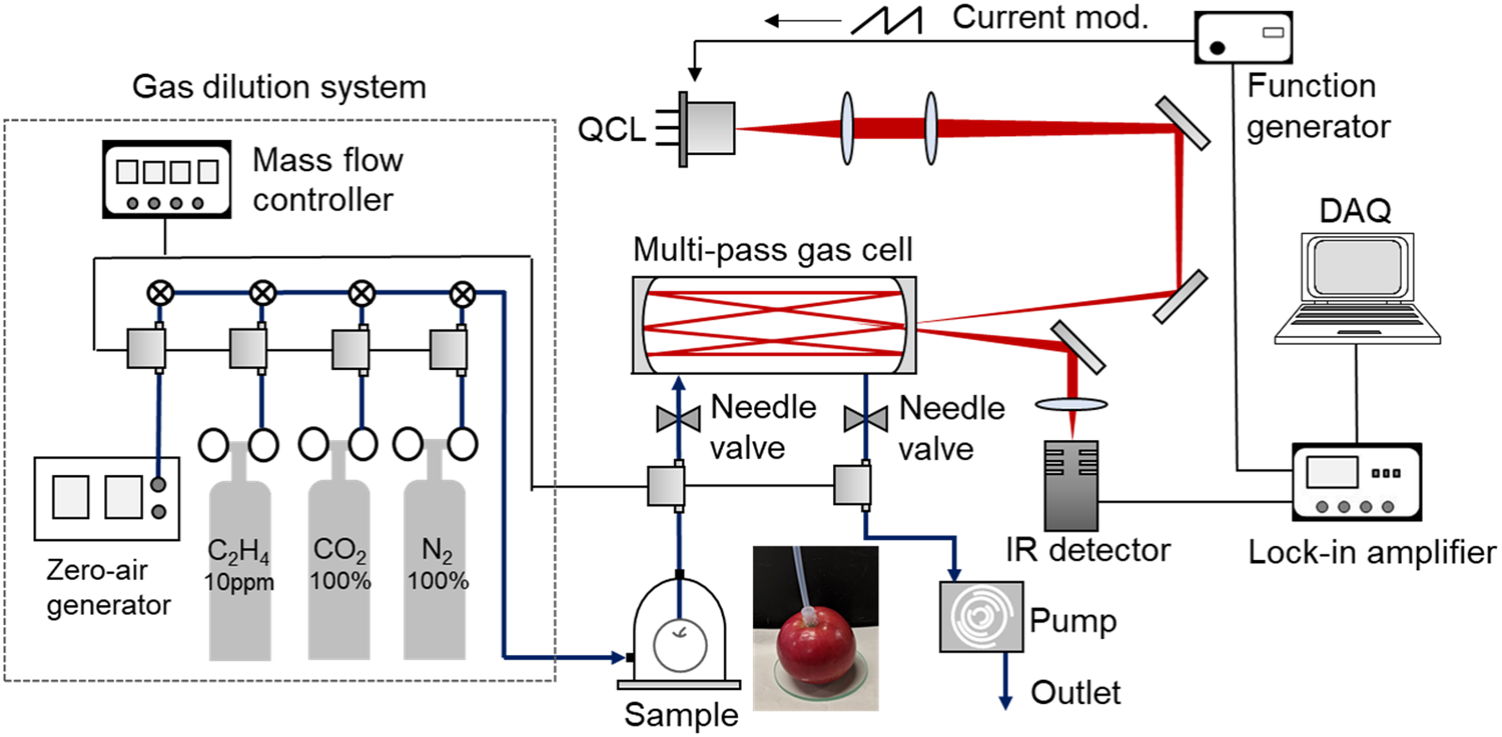
Schematic diagram of the C_2_H_4_ detection system. This system is based on LAS using the mid-IR QCL, the multi-pass gas cell, and the mid-IR detector.

Figure 1 shows a schematic diagram of the C_2_H_4_ detection system, which is mainly composed of the mid-IR QCL (QD10500CM1, Thorlabs Inc.), the astigmatic Herriott multi-pass gas cell (AMAC-76, Aerodyne Research), the mid-IR detector (PVI-4TE-10.6, VIGO system), and a gas dilution system. According to the HITRAN database, C_2_H_4_ has the strongest absorption lines at approximately 10.5 µm^27^. Therefore, we selected a single-frequency distributed feedback mid-IR QCL that oscillated at a central wavelength of 10.5 µm (~ 949.5 cm^−1^). The mid-IR QCL was operated in continuous wave mode, and the typical output power was 10 mW. The mid-IR QCL was controlled at 24 °C by integrated thermoelectric cooling, and the lasing wavelength was swept around the absorption peak at 10.5 µm by current modulation. The QCL output was passed through a lens pair and coupled to a multi-pass gas cell. The multi-pass gas cell provided a long optical pass length of 76 m to detect trace C_2_H_4_. The volume of the gas cell was 0.5 L. The output beam from the multi-pass gas cell was focused on the mid-IR detector. The inlet and outlet of the multi-pass gas cell were connected to the apples and scroll pump, respectively. By operating the dry scroll pump and simply making contact between the PTFE tube and the apple, the gas released from the apple was sampled in situ in an open environment without destroying the sample. By controlling the needle valves and mass flows installed at the inlet and outlet of the multi-pass gas cell, it was possible to perform LAS during constant flow of the sampled gas and while maintaining the internal pressure of the gas cell at an arbitrary pressure. By placing the apples in the glass jar connected to the gas dilution system, the atmospheric gas of the apple was also controllable. The gas dilution system was composed of mass flows, reference gases (N_2_, CO_2_, C_2_H_4_), and a zero-air generator. The CO_2_ and C_2_H_4_ concentrations were adjusted by controlling the flow rate of each reference gas and the zero-air generator. The spectral data were acquired on a PC with a time resolution of 1 s.

### Transmission spectra and detection limit

**Figure 2:**
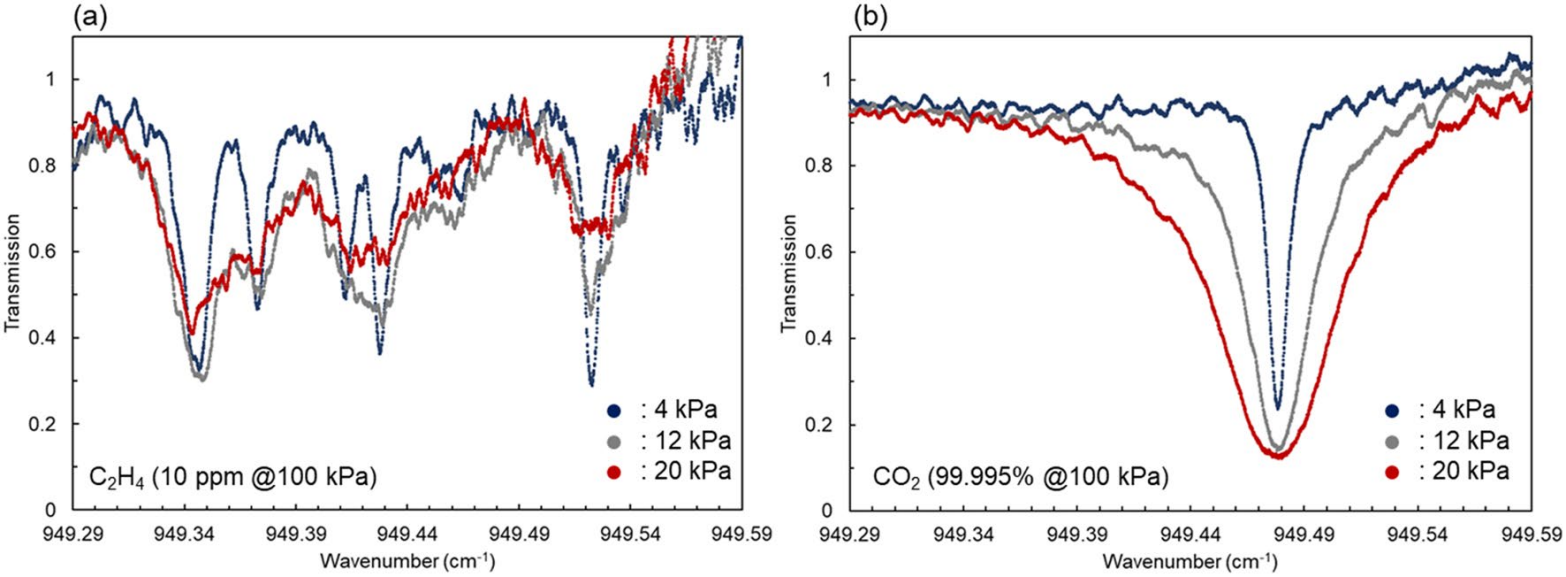
Transmission spectra of C_2_H_4_ and CO_2_. Blue, grey, and red lines show spectra at pressures of 4, 12, and 20 kPa, respectively, inside the gas cell. The concentrations of C_2_H_4_ and CO_2_ were 10 ppm and 99.995% at 100 kPa.

Figure 2 shows the results of the measured transmission spectra of C_2_H_4_ and CO_2_ using the C_2_H_4_ detection system. In this measurement, the multi-pass gas cell was directly connected to the gas dilution system without the apple to obtain the spectral data of the reference gases C_2_H_4_ and CO_2_. Here, the absorption intensity was weaker than that of C_2_H_4_, but the absorption spectrum of CO_2_ also appears near 949.48 cm^−1^. Therefore, this system was able to measure both the C_2_H_4_ and CO_2_ spectra. C_2_H_4_ and CO_2_ were supplied to the multi-pass gas cell from the gas dilution system at concentrations of 10 ppm and 99.995%, respectively, and the transmission spectra were measured while reducing the pressure inside the gas cell from 20 to 4 kPa. By reducing the internal pressure of the gas cell, the pressure broadening of the gas spectra was suppressed, and spectral narrowing of C_2_H_4_ and CO_2_ was observed. These results show that spectral measurement with pressure reduction helps prevent interference between C_2_H_4_ and CO_2_ absorption.

**Figure 3:**
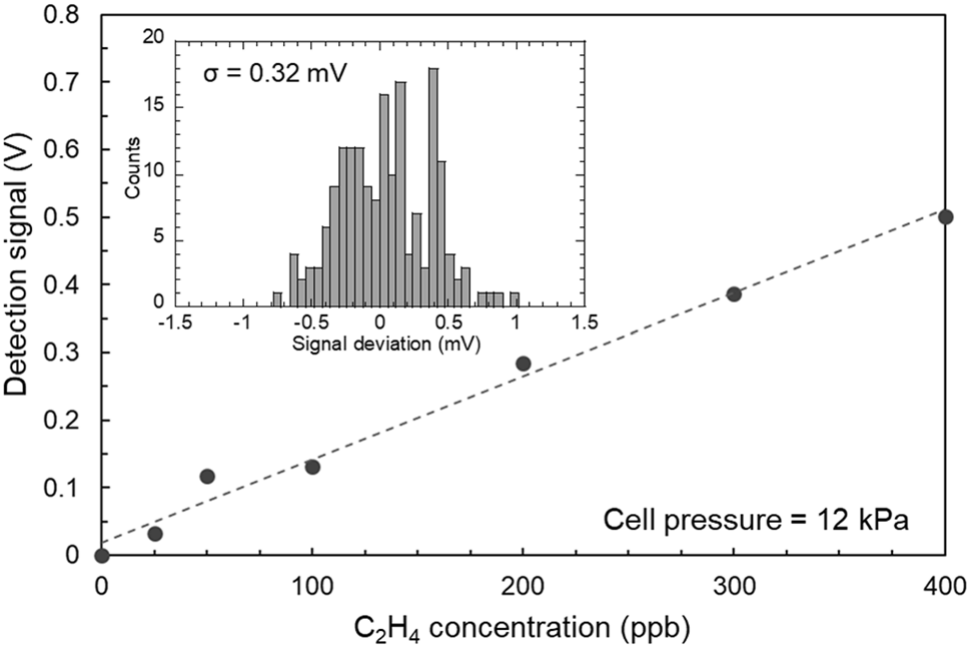
A detection signal of the mid-IR detector for different concentrations of C_2_H_4_. The pressure inside the gas cell was constant at 12 kPa. The inset shows the histogram plot of the detection signal using the N_2_ purged gas cell. The standard deviation (σ) was 0.32 mV.

Figure 3 shows the detection signal of the mid-IR detector for different concentrations of C_2_H_4_. Here, the value of the absorption peak at 949.35 cm^−1^ was used as the detection signal. The C_2_H_4_ concentrations were adjusted in the range of 25 ppb to 400 ppb by changing the mixing ratio of C_2_H_4_ (10 ppm) and N_2_ (99.999%) reference gases using a gas dilution system. The internal pressure of the gas cell was fixed at 12 kPa, and the measurement time was 3 min at each concentration. A linear fit to the experimental data yielded an R-square value of 0.9872 and an excellent linear response of the sensor. A histogram plot of the detection signal is shown in Fig. 3. The histogram plots were measured inside the gas cell purged with N_2_, and the standard deviation (σ) of the detection signal was 0.32 mV. Here, the noise equivalent absorption sensitivity (NEAS)^28,29^ of the C_2_H_4_ detection system was calculated as ${\text{NEAS}} = \Delta I/I \cdot L^{ - 1} \cdot f_{bw}^{ - 1/2}$, where $\Delta I/I$ is the 1σ value of the limiting noise level, $L$ is the pass length of the gas cell, and $f_{bw}$ is the detection bandwidth. The $\Delta I/I$ value of 0.36% was obtained from the histogram plot, so that the NEAS was ~ 1.5 × 10^−8^ cm^−1^ Hz^−1/2^ ($L = 76\;{\text{m}},\;f_{bw} = 1\;{\text{kHz}}$). In addition, the detection limit was estimated using three-standard deviations (3σ)^30^. The 3σ value of 0.96 mV was obtained from the form histogram plot, which corresponded to 0.8 ppb when compared with the linear fitting line in Fig. 3. However, in the lower concentration region (< 100 ppb), some measurement points deviated from the linear fitting line. When supplying low concentration C_2_H_4_ using the gas dilution system, ​the mass flow meter and pressure gauge have to read out a lower flow rate and pressure. Therefore, in the low concentration region, as compared with the high concentration region, the effective measurement accuracies of the mass flow meter and pressure gauge were lowered, which caused the deviation in the low concentration region. The effective quantification limit was estimated to be approximately 2.8 ppb, which was equivalent to ten-standard deviations (10σ)^30^. The detection ability of ppb-level C_2_H_4_ was sufficient to apply this system to the analysis of C_2_H_4_ released from plants.

### Non-destructive and in-situ sampling of C_2_H_4_ released from the apples

**Figure 4:**
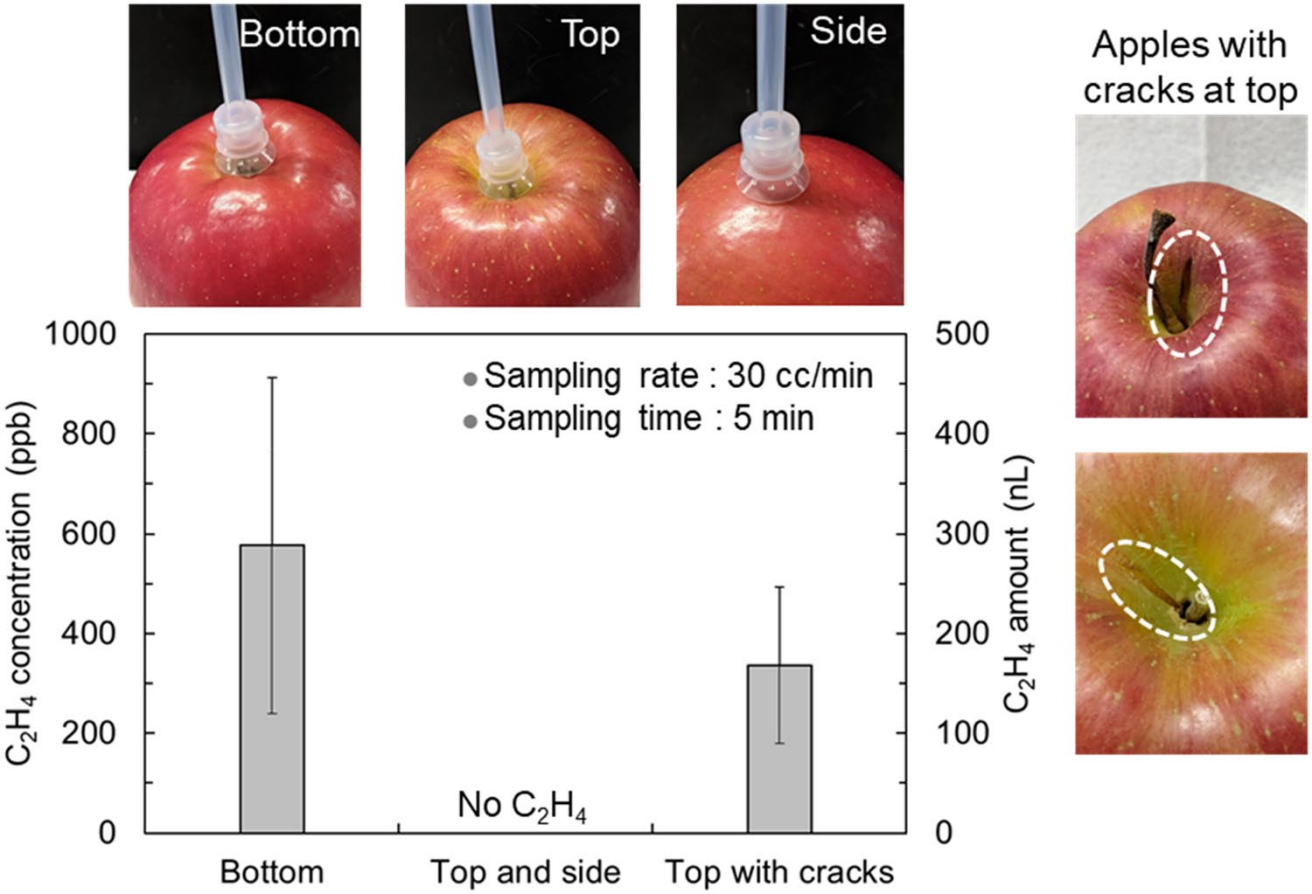
Location dependence of C_2_H_4_ concentrations and amount released from apples. Each of the ten apples was sampled in situ to detect released gas at the top and bottom of the core, and the side surface. Five apples released C_2_H_4_ from the bottom, and the two apples with a crack on their top released C_2_H_4_ from the top. No C_2_H_4_ was detected from anywhere in the remaining three apples.

We have demonstrated trace C_2_H_4_ detection released from apples by non-destructive and in-situ gas sampling in an open environment. Apples are typical climacteric fruits, characterised by an increase in C_2_H_4_ generation and respiration during ripening. In this test, the cultivar ‘Fuji’ was used to clearly detect the source of C_2_H_4_ generation in the apple, because it is known to produce remarkably low levels of ethylene gas^31,32^. Figure 4 shows the locations of C_2_H_4_ concentrations. The gases were sampled in situ at the top and bottom of the apple cores and side surfaces of the apples. The sampling flow rate and time were 30 cc/min and 5 min, respectively, and the internal pressure of the gas cell was 12 kPa. Ten apples were used for the measurements. Error bars represent standard deviation. Two apples had cracks on the top of the core, and the other eight apples had no visible cracks. The cracks were from existing natural injuries; they were not intentionally made for the experiment. When using the cracked apples as samples, a C_2_H_4_ concentration of 340 ± 160 ppb was detected only at the top of the core. Five of the eight apples without cracks released C_2_H_4_ at a concentration of 580 ± 360 ppb from the bottom. In the three remaining apples, C_2_H_4_ was not detected in any position. No C_2_H_4_ was observed on the side surfaces of any apples. The reason for the absence of C_2_H_4_ detection may be that C_2_H_4_ released from the side surface was at a much lower concentration than the detection limit. C_2_H_4_ release at ~ 100 ppb concentration was observed from the side surface of a few apples when using the PTFE tube with a sucking disk at the tip for gas sampling. However, in this case, the sucking disk quickly sticks to the apple surface, and the apples become stressed by forced gas suction. This sampling method was not stress-free. Additionally, the method is not effective when using plants with bumpy surfaces as samples. Based on the above results, apples without cracks were used as samples in subsequent experiments, and the gas was sampled from the bottom of the core without the sucking disk.

**Figure 5:**
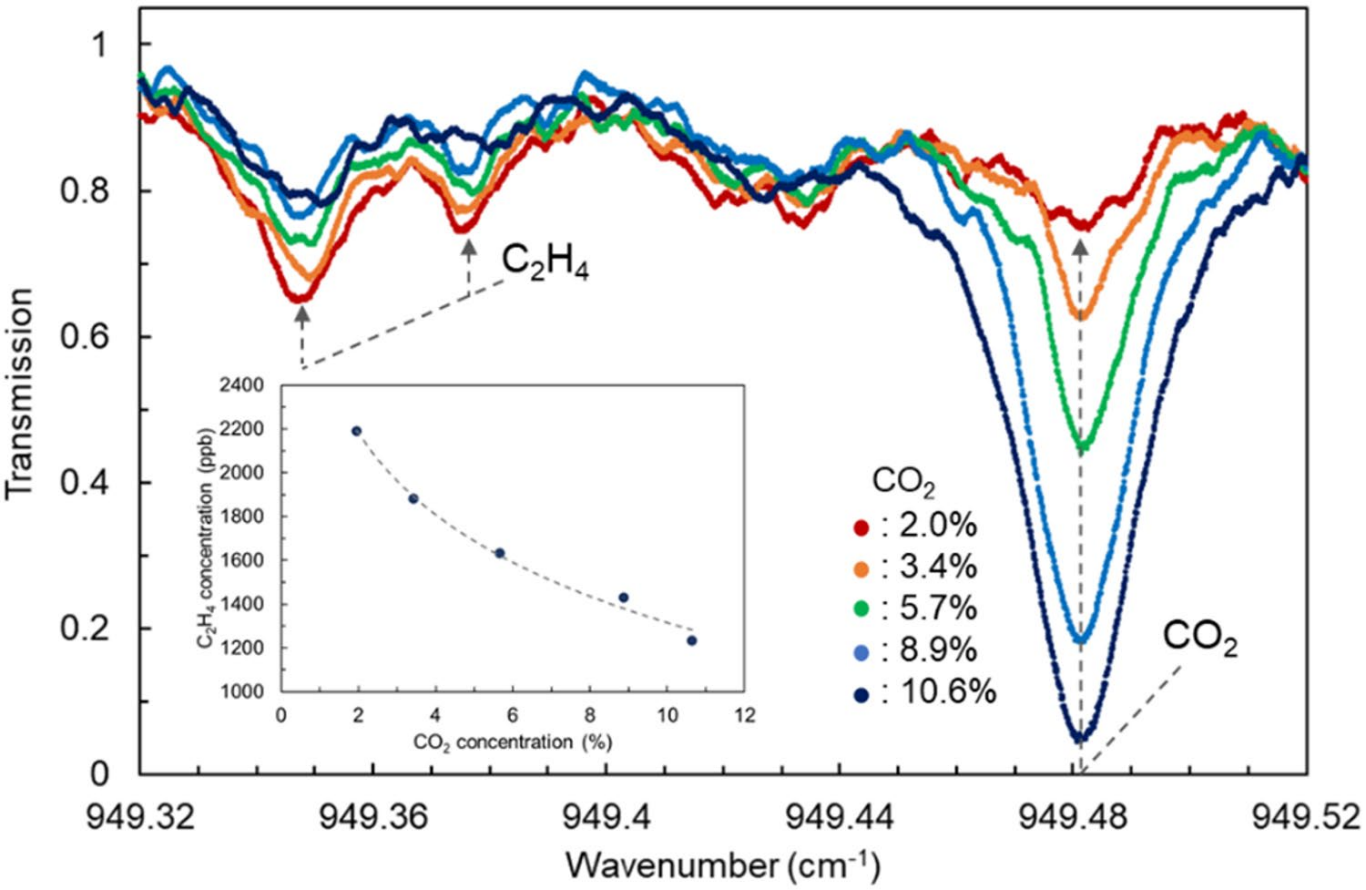
The transmittance spectra of gases from apples. Each spectrum shows the changes in the amount of C_2_H_4_ released with changing the concentration of CO_2_ in the atmosphere gas of apples. CO_2_ concentrations were controlled at 2.0, 3.4, 5.7, 8.9, and 10.6% using the gas dilution system. The inset shows the relationship between C_2_H_4_ and CO_2_ concentrations.

Figure 5 shows the transmittance spectra of C_2_H_4_ and CO_2_ with changing concentrations of CO_2_ in the atmospheric gas of the apples. The apples were placed in a glass jar, and the concentration of CO_2_ supplied to the glass jar was controlled between 2.0 and 10.6% using a gas dilution system. The mid-IR absorptions of both C_2_H_4_ and CO_2_ were observed simultaneously in the wavelength region of 949.32–949.52 cm^−1^. As the CO_2_ concentration increases from 2.0 to 10.6%, CO_2_ transmission near 949.48 cm^−1^ decreases from 75 to 5%, which indicates that CO_2_ absorption has increased. At the same time, an increase in C_2_H_4_ transmission around 949.35 cm^−1^ was observed. This result shows that C_2_H_4_ release from apples decreases as the atmospheric CO_2_ concentration of the apple increases. It was confirmed that the C_2_H_4_ concentration decreased from 2200 to 1200 ppb as the CO_2_ concentration increased from 2.0 to 10.6%. Using our C_2_H_4_ detection system, we succeeded in visualising the suppression of C_2_H_4_ production by CO_2_ treatment, which is a commonly used controlled atmosphere storage method^32,33^. The same phenomenon was observed in another cultivar termed “Kiou” (data not shown).

### Real-time detection of C_2_H_4_

**Figure 6:**
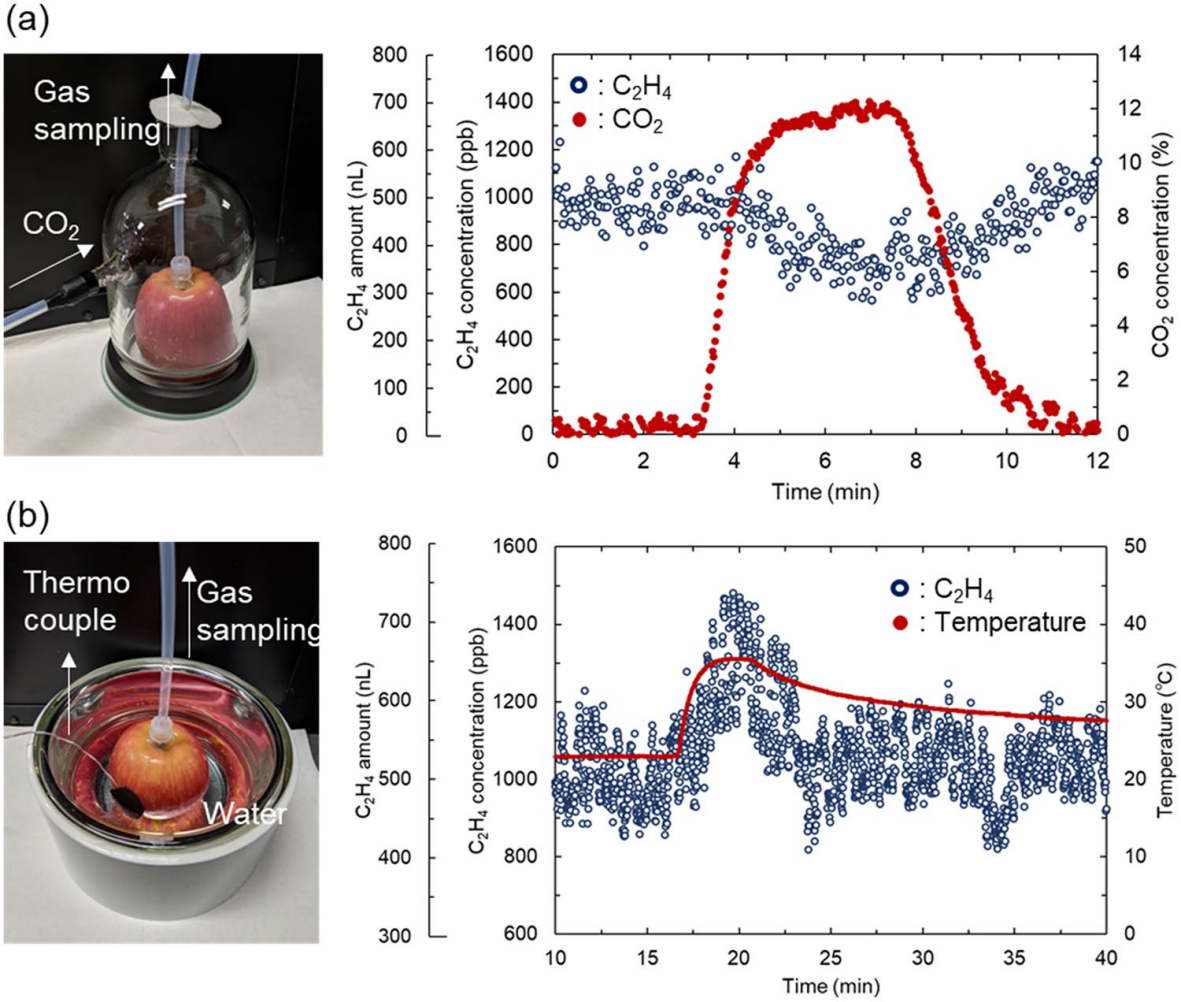
Real-time detection of C_2_H_4_ released from apples with CO_2_ concentration change (**a**) and the temperature change the of apple surface (**b**). Blue dots show the changes in C_2_H_4_ concentration and amount released from the apple in the gas cell. The red dots in (**a**) and (**b**) represent CO_2_ concentrations around the apple and apple surface temperature, respectively.

Figure 6 shows the results of real-time monitoring of how the C_2_H_4_ concentration released by the apple changes with the environmental response. As shown in Fig. 6a, the C_2_H_4_ concentration was measured in real time by changing the atmospheric CO_2_ concentration of the apple. The experimental setup was the same as that in Fig. 5. The CO_2_ concentration supplied to the glass jar containing the apple was controlled using a gas dilution system. The C_2_H_4_ concentration decreased from 1000 to 700 ppb as the CO_2_ concentration increased to 12%. Then, it returned to 1000 ppb when the CO_2_ concentration returned to atmospheric concentration. It was observed in real-time that the change in C_2_H_4_ concentration was inversely correlated with the change in CO_2_ concentration. Figure 6b shows the change in the C_2_H_4_ concentration when the apple was heated. The apple was soaked in a Dewar vessel filled with water at 22 °C, and a thermocouple for temperature measurement was attached to the apple surface. When the temperature was rapidly increased to 35.5 °C by adding boiling water, the C_2_H_4_ concentration increased from 1000 to 1500 ppb. As the temperature decreased, the C_2_H_4_ concentration also decreased. We observed for the first time that the C_2_H_4_ concentration released from apples changed with a time constant of several minutes in response to environmental changes.

### C_2_H_4_ identification by TD-GC/MS and bioassay

**Figure 7:**
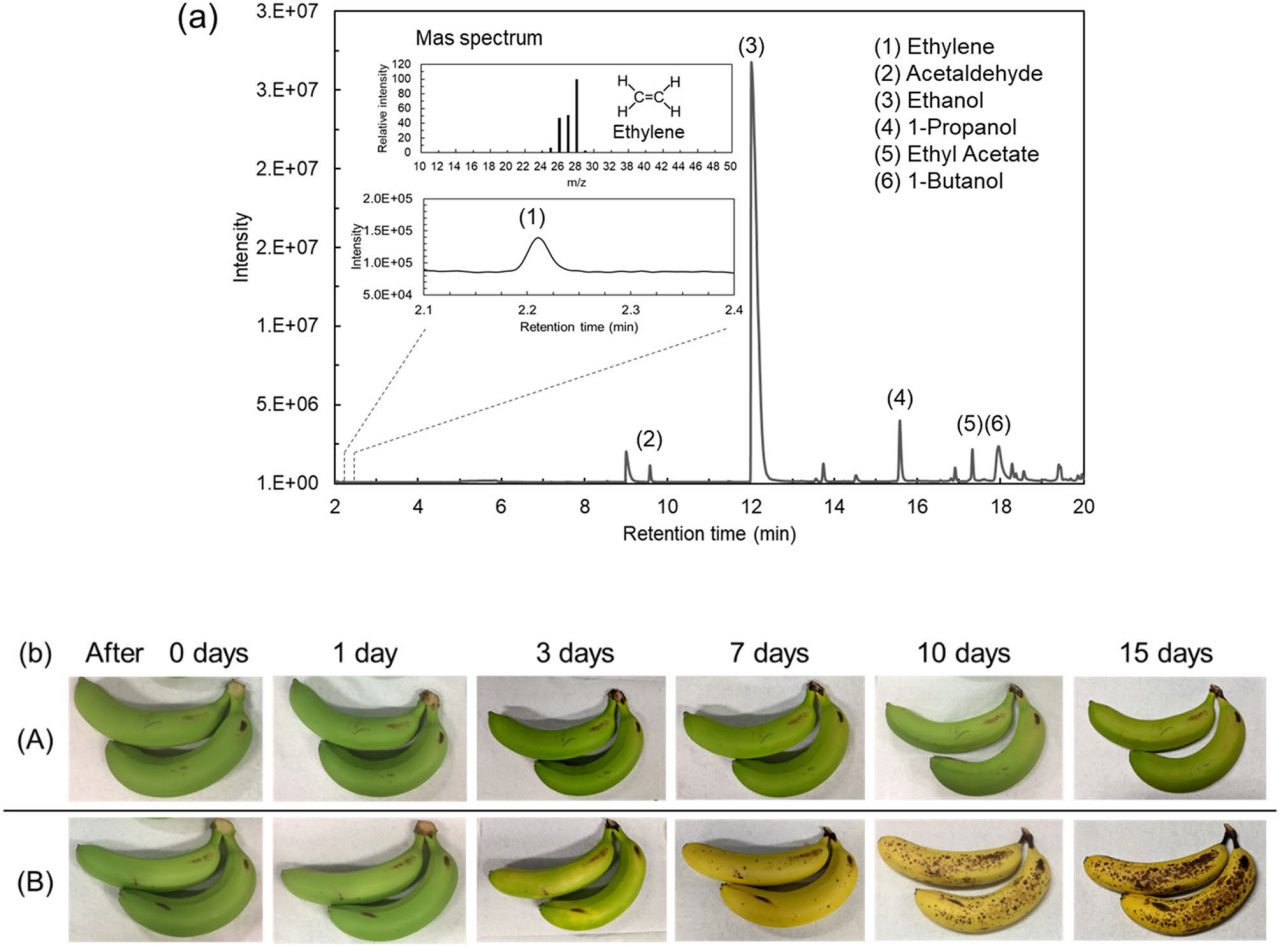
C_2_H_4_ identified in sample gas released from an apple. (**a**) Chromatograms of TD-GC/MS analysis for the sample gas. The insets show the C_2_H_4_ chromatogram peak at 2.2 min retention time and the mass spectrum of the C_2_H_4_. (**b**) Bioassay of banana ripening. Group (A): bananas without exposure to the gas released from apples. Group (B): bananas with exposure to the gas released from an apple over 24 h.

We confirmed the existence of C_2_H_4_ in the gas released from the apple by thermal desorption gas chromatography-mass spectroscopy (TD-GC/MS) and bioassays. Adsorbents (MonoTrap RGPS TD, GL Science) were inserted inside the PTFE tube while sampling the apple-released gas to prepare analytical samples for TD-GC/MS. The flow rate in the PTFE tube was 30 cc/min, and the time for the gas to flow through the tube was 90 min. The chromatogram of the gas released from the apples is shown in Fig. 7a. The sampled gas contained various VOCs (e.g., ethylene, acetaldehyde, and ethanol), and each peak identification of the VOCs is shown in Fig. 7a. The peak retention time of C_2_H_4_ was observed at 2.2 min. The mass spectrum at 2.2 min shows good agreement with that of C_2_H_4_.

Next, we demonstrated a banana ripening test as a bioassay using the sample gas, because bananas are also typical climacteric fruits. Figure 7b shows the results of ripening bananas with apple-releasing gas collected by in-situ sampling. We used unripened green bananas as the samples. Gas sampled in situ at a flow rate of 30 cc/min from the apple was allowed into the gas chamber at a volume of 2 L, and the green banana was placed in the glass chamber and exposed to the sampling gas for 24 h. After sample gas exposure, the green banana was removed from the glass chamber, and the ripening process was compared with another green banana which was not exposed to the sample gas. A comparative test was performed at room temperature. As a result, a clear visual difference between both bananas was observable on the third day, confirming that bananas exposed to gas released from the apple ripened faster than the other bananas. This result shows that C_2_H_4_ in the apple-releasing gas causes banana ripening by inducing climacteric rise^6,34–36^. The TD-GC/MS and banana ripening tests showed that the gases from the apple we sampled contained C_2_H_4_. We confirmed that our C_2_H_4_ detection system is effective for the non-destructive real-time monitoring of trace C_2_H_4_ released from apples.

## Conclusions

In this study, we succeeded in real-time monitoring of C_2_H_4_ concentration changes in gas released from apples by non-destructive and in-situ gas sampling in an open environment. Monitoring was demonstrated using an original C_2_H_4_ detection system based on LAS. We also demonstrated the rapid concentration changes of C_2_H_4_ released from apples when apples were stressed by changes in atmospheric gas concentration or surface temperature. Furthermore, a special sampling chamber or bag is not required, which provides a great advantage in that gas can be sampled from an arbitrary place of fruit. Gas sampling from wounds, lesions caused by diseases and pests, and other localised areas of plants are possible. We expect that our method will allow real-time visualisation of C_2_H_4_ dynamics following changes in environmental response. By changing the lasers that make up this gas detection system, various VOCs other than C_2_H_4_ can be detected. More detailed information about the plant gas response can be obtained by combining it with conventional GCMS research. Our results show promise in advancing analytical techniques for plant environmental responses.

## Methods

### Plant materials

We purchased ‘Fuji’ apples (LOPIA Co. Ltd, Saitama, Japan) and used the same cultivar for all experiments and analyses. For the bioassay of banana ripening, we used green bananas (‘Cavendish’) without exposure to C_2_H_4_. Green bananas were purchased from Konmatsu Co., Ltd. (Iwate, Japan). During the experiment, both the apples and bananas were stored at 20–22 °C.

### Reference gas preparation and internal pressure control of multi-pass gas cell

We prepared three reference gases and a zero-air generator (Zero Air Supply Model-111, Thermo Fisher Scientific, Waltham, MA, USA) for the gas dilution system. The three reference gases were C_2_H_4_ (10 ppm), G1-CO_2_ (99.995%), and G1-N_2_ (99.99995%), and all gases were charged in 10 L high-pressure gas cylinders. According to the manufacturer specifications, the zero-air generator provides dry air without NOx, SO_2_, O_3_, CO, and hydrocarbons. All gas supply lines had mass flow meters (Standard Mass Flow Controller MODEL 3660 SERIES, KOFLOC, Kyoto, Japan), and the flow rate could be controlled. By changing the mixing ratio with N_2_ or zero air, C_2_H_4_ and CO_2_ can be diluted to any concentration. The reference and sampling gases were introduced into the multi-pass gas cell using a dry scroll pump connected to the gas cell. Then, using a needle valve attached to the inlet and outlet of the gas cell, the internal pressure of the gas cell can be adjusted between 2 and 100 kPa while maintaining a constant flow rate in the gas cell.

### Thermal desorption-gas chromatography/mass spectrometry (TD-GC/MS)

We used thermal desorption gas chromatography-mass spectroscopy (TD-GC/MS) to analyse numerous VOCs present in the apple-releasing gas. The TD-GC/MS consisted of a GC/MC (GCMS-TQ8040, Shimadzu, Kyoto, Japan) and a high-performance multimode injector (OPTIC-4, Shimadzu). For the thermal desorption analysis, we used MonoTrap RGPS TD (GL Science) as the absorbent. The adsorbents were inserted into a glass tube (MonoTrap TD Liner for OPTIC/LINEX) and introduced into the injector port for thermal desorption. The initial temperature of the injector port was set at 40 °C, and then the temperature was increased to 230 °C at a rate of 10 °C/s to desorb the VOCs in splitless mode. The desorbed VOCs were concentrated at the headspace of the GC column using cryofocus trapping cooled to − 150 °C. After cryofocus trapping, the GC oven temperature was maintained at 35 °C for 3 min, increased to 200 °C (rising rate = 10 °C/min), and then held at 200 °C for 2 min. An Rt-Q-BOND column (ID = 0.32 mm, length = 30 m, df = 10 μm, RESTEK) was used to separate the eluting compounds, and the carrier gas was high purity He (99.9999%) with a constant flow of 5 ml/min. The MS detector was operated in scan mode from 20 to 300 amu at 200 °C for electron ionisation. C_2_H_4_ identification was achieved by comparing the mass spectra and retention times with the reference gases of C_2_H_4_. We used GCMSsolution software (Ver. 4.45) and Mass Spectral library (NIST 14) to analyse the retention time and mass spectra, respectively. In this measurement, an apple confirmed to absorption spectral peak of C_2_H_4_ by our C_2_H_4_ detection system was used as a sample. The thermal desorption analysis using the absorbent was carried out three times and the C_2_H_4_ was detected in all measurements.
